# FT-IR/ATR Solid Film Formation: Qualitative and Quantitative Analysis of a Piperacillin-Tazobactam Formulation

**DOI:** 10.3390/molecules25246051

**Published:** 2020-12-21

**Authors:** Ioanna Chrisikou, Malvina Orkoula, Christos Kontoyannis

**Affiliations:** 1 Department of Pharmacy, University of Patras, University Campus, GR-26504 Rio Achaias, Greece; ioannach94@gmail.com (I.C.); malbie@upatras.gr (M.O.); 2 Institute of Chemical Engineering Sciences, Foundation of Research and Technology-Hellas (ICE-HT/FORTH), GR-26504 Platani Achaias, Greece

**Keywords:** FT-IR/ATR, sample preparation, film formation, piperacillin, tazobactam

## Abstract

FT-IR/ATR analytical technique is one of the most applicable techniques worldwide. It is closely associated with easy-to-use equipment, rapid analysis, and reliable results. This study reports the simultaneous qualitative and quantitative analysis of two active pharmaceutical ingredients (APIs), of a piperacillin and tazobactam formulation using a film formation method. This method requires film formation on the ATR crystal, resulting from solvent evaporation of a small amount of liquid sample. Good contact between the film and the crystal led to the identification of both APIs, although tazobactam was of low content in the formulation mixture. The quantification of the APIs in the commercial mixture was also achieved, using a single calibration line with a correlation coefficient equal to 0.999, not only after film formation but also in the initial dry formulation before reconstitution. The present spectroscopic technique combined with the proposed relatively simple sample treatment outweighs chromatographic protocols already applied, which require specialized staff and are costly, time-consuming, and not environmentally friendly. Taking all the above into consideration, it turns out that such an approach has the potential to be used for off-line quality control procedures in manufacture or, in terms of portable equipment and automated software, anywhere for on-site analysis, even in a hospital workflow.

## 1. Introduction

Fourier Transform Infrared Spectroscopy (FT-IR) is a well-established analytical technique. Mid-IR region (4000–600 cm^−1^) offers the ‘’fingerprint’’ of the analyte, as it is rich in information about the structure of the functional groups of the sample tested [1]. Apart from that, it can be applied for quantitative analysis as the energy absorbed at a particular wavelength is in proportion with the number of bonds absorbing the associated quanta of energy [2]. FT-IR/ATR (Attenuated Total Reflectance), based on internal total reflectance, constitutes an alternative mode of the customary technique, which has numerous applications in recent years in a variety of fields including medicine, food industry, environmental, and forensic science [3,4,5,6,7]. In a typical ATR experiment, the incident infrared radiation interacts with the analyte molecules on the crystal surface via an evanescent wave created by total reflectance in the crystal. The attenuation of the evanescent field by the absorption in the sample is then detected. Since this field decays exponentially the depth of penetration in the sample corresponds to few microns (usually 1–2 um) [1,2]. Worth mentioning is the fact that the quality of the spectra obtained, depends strongly on procedures like sample preparation and handling. The good contact between the sample and the crystal surface plays a vital role in excluding the possibility of a noisy spectrum acquisition. The quality of the obtained spectra in the case of solids or powders depends on the applied downforce, whereas when handling liquid samples, the determinant factor is the number of analyte molecules being in contact with the surface of the crystal, so as the solvent contribution to be minimized [7]. In the pharmaceutical field, ATR has been used for qualitative as well as quantitative analysis of either solid or liquid samples. Graham Lawson and his team used this technique to develop a rapid method of quantifying counterfeit tablet formulations. Their method was developed without the need of solvents as the samples under test were in the form of powder or tablets that had been crushed prior to analysis [8]. Kerry J. Hartauer and J. Keith Guillory suggested an alternative procedure for the simultaneous determination of trimethoprim and sulfamethoxazole in a formulation intended for intravenous administration. Their method insisted that the sample solutions be poured into a Circle Cell to cover the instrument crystal completely and 5 min of purge to be allowed before recording the spectra [9]. The percentage of methamphetamine contained in illicit drug mixtures was determined rapidly and effectively without any significant pre-preparation required [10]. Besides that, Alaa A. Makki and his team applied ATR spectroscopy for the individual analysis of three commercially available anticancer drugs, TEVA^®^, MYLAN^®^ and CERUBIDINE^®^, respectively containing doxorubicin, epirubicin, and daunorubicin. This study was conducted in order for ATR spectroscopy to be assessed as a tool for the analysis of therapeutic solutions in a hospital workflow. All drug samples measured were in liquid form, while the pre-measurement sample treatment included the film formation of a small (2 µL) sample volume on the instrument crystal through solvent evaporation [11]. Although film formation technique has been reported as an ATR sample preparation method for the analysis of active pharmaceutical ingredients in formulations, there is no published application of such a technique for not only the identification but also the quantification of two different APIs in a single formulation being initially in its solid form. This paper reports on the employment of the film-formation, preparation technique for the qualitative and quantitative analysis of a piperacillin (PIP) and tazobactam (TAZ) intravenous formulation with a mass ratio of APIs equal to 8:1 (89% PIP-11% TAZ). Piperacillin is a broad-spectrum b-lactam antibiotic, which inhibits the synthesis of the bacterial cell wall [12,13], while tazobactam is a beta-lactamase inhibitor, which increases and expands the antimicrobial spectrum of piperacillin. In particular, it protects the antibiotic from degradation caused by beta-lactamase enzymes [12,14]. Up to date, in most cases, PIP and TAZ in formulations have been simultaneously analyzed through exploitation of time-consuming LC (liquid chromatography) protocols [15,16,17], a procedure that is required by pharmacopoeia for the identification of each API and its impurities [18]. Ultra high performance liquid chromatography tandem mass spectrometry (UHPLC-MS/MS) has also been applied for the simultaneous determination of the two APIs, not in the formulation but in different biological matrices (serum, urine, renal replacement therapy effluent) in regards to therapeutic drug monitoring (TDM) after administration [19,20]. Although this method requires microsample volumes and the run time does not exceed 5 min, the analytical procedure requires more expensive equipment and more specialized staff and cannot be applied directly to the as-received initial dry formulation, before reconstitution. UV/Vis spectroscopy has also been used, but its application proved to be complex since it requires selection of appropriate derivative order and smoothing factor [21,22].

## 2. Results

### 2.1. Identification of PIP and TAZ

#### 2.1.1. Powder Spectra

**Figure 1:**
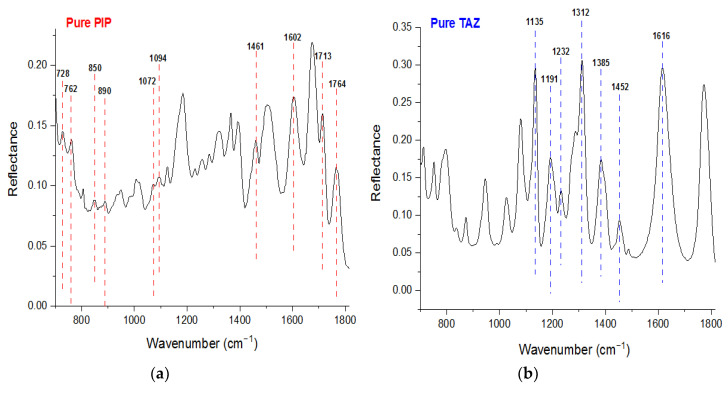
(**a**) Quantitative Fourier Transform-Infrared/Attenuated Total Reflectance (FT-IR/ATR) powder spectrum of pure piperacillin (PIP) and (**b**) FT-IR/ATR powder spectrum of pure tazobactam (TAZ).

Initially, FT-IR/ATR spectra of the powder of both the pure active substances were obtained (Figure 1) and some of the most significant peaks of them were attributed to the vibration modes of their molecules (Table 1) [1].

**Figure 2:**
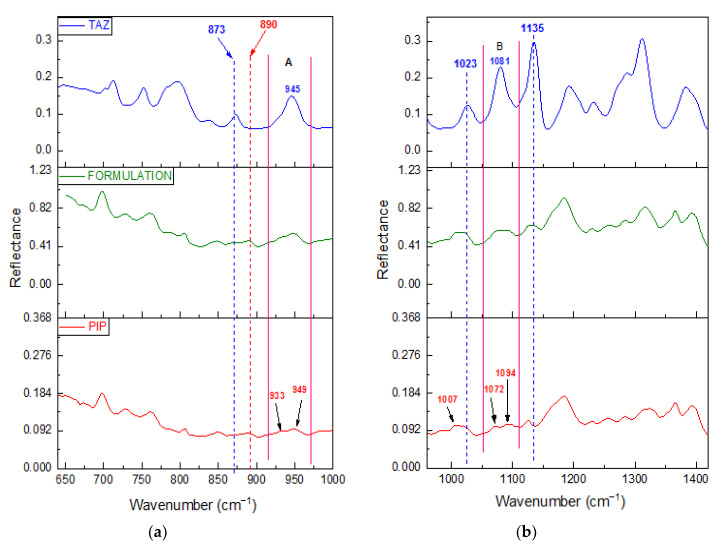
FT-IR/ATR Powder Spectra of PIP, TAZ, and the formulation: (**a**) from 640 to 1000 cm^−1^ and (**b**) from 960 to 1420 cm^−1^. Differences between PIP and formulation spectra due to TAZ presence in the commercial mixture are with blue dotted lines and in the spectral regions A (915–971 cm^−1^) and B (1052–1111 cm^−1^). The characteristic peak of PIP at 890 cm^−1^ is with the red dotted line.

The FT-IR/ATR spectrum of the formulation was then obtained. A great similarity between the spectrum of the commercial mixture and that of piperacillin was observed, as shown in Figure 2. This was expected due to the high content of piperacillin in the formulation.

On the other hand, TAZ peaks are extensively overlapped by those of PIP, and the presence of TAZ in the formulation becomes evident through small differences between formulation and PIP spectra, such as strengthening of PIP peaks at certain positions or change in intensity ratio between neighboring PIP peaks. All these observations are summarized in Table 2.

#### 2.1.2. Film Spectra

Subsequently, solutions of pure PIP (50 mg/mL), TAZ (30 mg/mL) and the formulation (44.44 mg PIP/mL, 5.56 mg TAZ/mL) were prepared and an aliquot, equal to 5 µL was placed on ATR crystal. Spectra were obtained 20 min later from the film formed thereon after evaporation of the solvent. Worth mentioning is the fact that the concentration of each solution was of such a value that adequate sample quantity could be in good contact with ATR crystal after solvent had been evaporated and a satisfying signal to noise ratio could be obtained.

**Figure 3:**
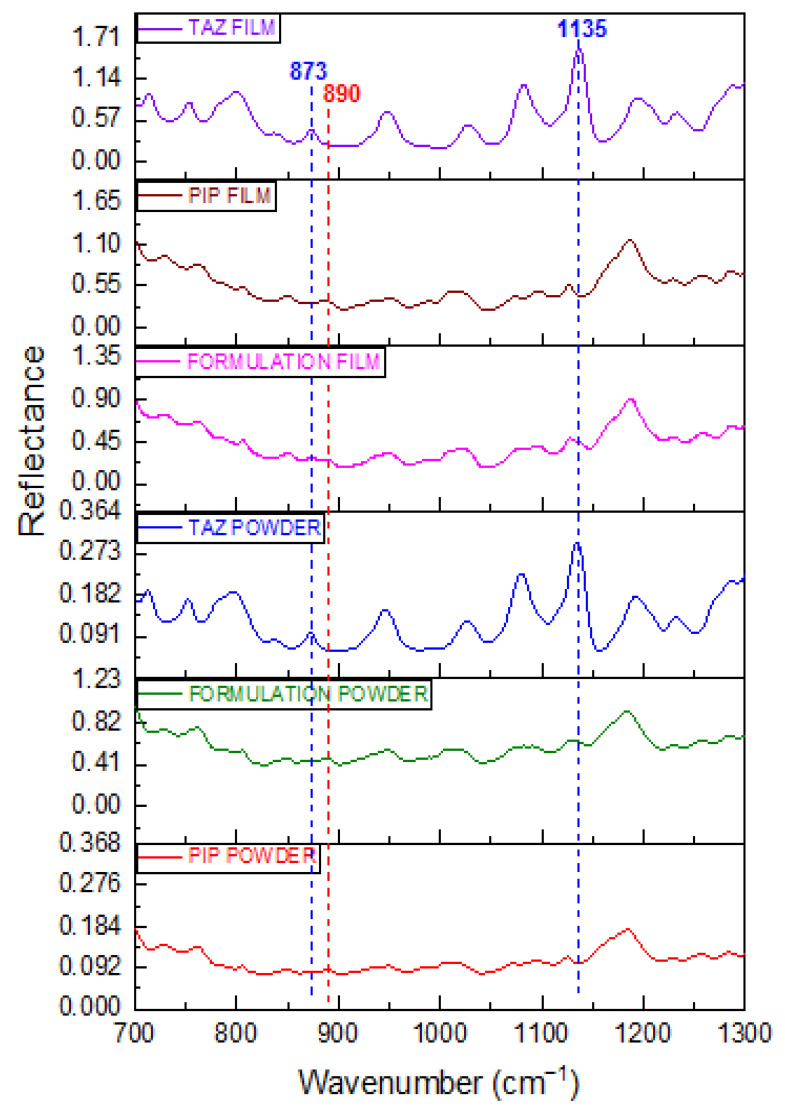
FT-IR/ATR spectra of PIP, TAZ, and Formulation Film (brown, purple, and pink line respectively), as well as that of PIP, TAZ, and Formulation Powder (red, blue, and green line respectively) at spectral region from 700 to 1300 cm^−1^.

Film spectra found to be of better quality than those of the powder. The term better quality is referred to the higher intensity of the peaks detected in spectra obtained from the film, as well as the better signal-to-noise ratio. This is achieved due to the better contact between the analyte and the ATR crystal. Apart from that, no peak shift is observed in the case of the film spectra, in comparison with the respective spectra obtained from the powder of the substance under test. This is valid for both PIP and TAZ and means that after solvent evaporation, no other polymorph or hydrated form of the active substances is precipitated. Figure 3 is the proof of the two previous conclusions.

Emphasis should be placed on peaks at 873 and 1135 cm^−1^, which are the main evidence of tazobactam presence in the formulation, since non-overlapping characteristic peaks of the API do not exist. Particularly, either the strengthening of PIP peak at 873 cm^−1^ or the shoulder detected at 1135 cm^−1^, due to TAZ, are much more distinct in case of film spectra acquisition. Thus, identification of TAZ API is much easier. 

### 2.2. Quantification of PIP and TAZ

**Figure 4:**
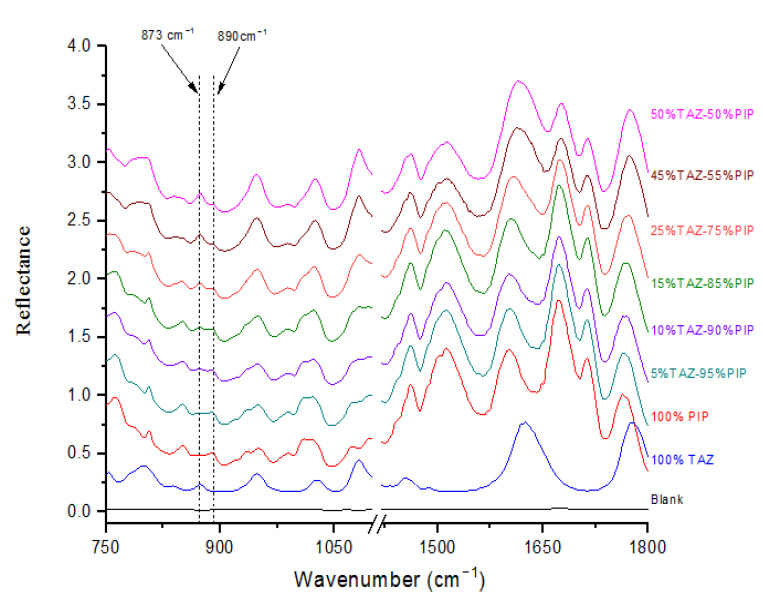
FT-IR/ATR Film Spectra obtained from solutions of standard solid mixtures with a mass ratio of PIP-TAZ equal to 50% ΤAΖ-50% PIP (pink line), 45% TAZ-55% PIP (brown line), 25% TAZ-75% PIP (orange line), 15% TAZ-85% PIP (green line), 10% TAZ-90% PIP (purple line) and 5% TAZ-95% PIP (navy line). Additionally, ATR Film Spectra obtained from pure PIP (red line), pure TAZ (blue line) and blank solution (water film, black line). Note that the spectra have been shifted along y axis for clarity.

Solutions of a wide range of standard PIP-TAZ mixtures were prepared and their ATR spectra were obtained, using the film formation technique (Figure 4). The purpose of that procedure was the construction of a calibration line for the quantification of % *w*/*w* content of each API in the solid formulation. Mass ratio of the standard mixtures prepared, ranged from 5% TAZ-95% PIP to 50% TAZ-50% PIP, taking into account that the composition of the commercially available formulations is 11% TAZ-89% PIP.

As TAZ concentration in the solid mixture increases, the peak at 873 cm^−1^ becomes gradually more evident and it ends up outweighing its neighboring peak at 890 cm^−1^.

The mean peak height ratio, *I*(*873*)/*I*(*890*) and relative standard deviation (RSD) values resulting from three replicate measurements were calculated for each standard (Table 3).

At this point, low RSD values in Table 3 are indicative of the reproducibility of replicate sample measurements as well as the high sample homogeneity achieved, given that the instrumental conditions remained constant. 

**Figure 5:**
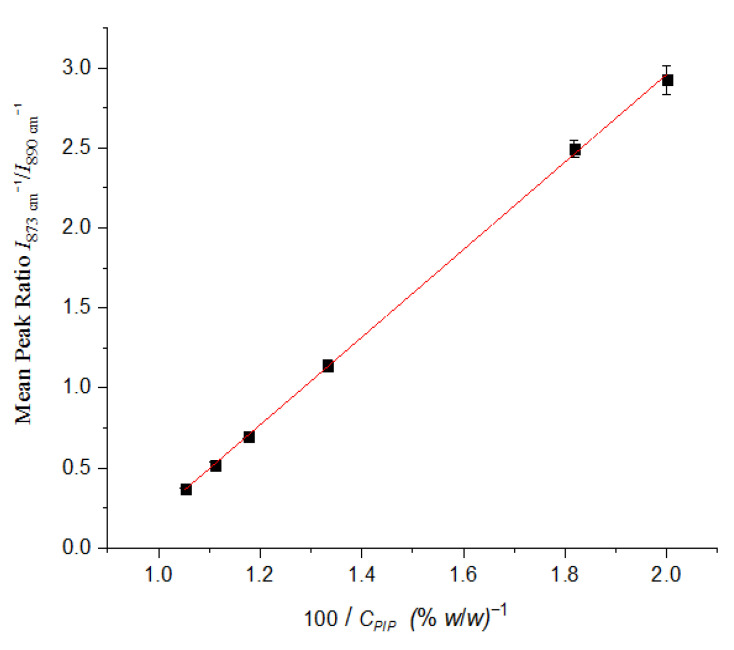
Calibration line for both PIP and TAZ for solid mixtures of them, with APIs’ mass ratio ranging from 5% TAZ-95% PIP to 50% TAZ-50% PIP.

The calibration graph plotted is reproduced in Figure 5 and was constructed through least square regression method, using the ratio $\frac{I\left(873\right)}{I\left(890\right)}$ (y axis) against ratio $\frac{100}{C_{PIP}}$ (x axis) [See Appendix A].

The equation describing the calibration line was:

(1) $$\frac{I\;\left(873\;{\mathrm{cm}}^{-1}\right)}{I\;\left(890\;{\mathrm{cm}}^{-1}\right)}\;=\;(2.74\;\pm \;0.024)\;\times \;\left(\frac{100}{C_{PIP}}\right)\;+\;(-2.52\;\pm \;0.026),\;(R^{2}\;=\;0.999),$$

Since minimum amount of tazobactam corresponds to maximum amount of piperacillin, the limit of detection (LOD) of tazobactam was calculated after many blank (PIP film) measurements. Using Equation (1), LOD was found to be 1.618% *w*/*w* and LOQ (limit of quantification) equal to 4.854% *w*/*w*. LOD value was confirmed by visual evaluation method [23].

In order to test the performance of the calibration line a solution of 11% TAZ-89% PIP was treated as unknown sample. The PIP concentration was found to be (89.37 ± 0.40)% *w*/*w* and that of TAZ was equal to (10.63 ± 0.40)% *w*/*w*. The relative error values of the results mentioned above were found to be 0.41% and 3.33%, respectively. 

In order to test if the calibration line could be applied directly to solid formulation before reconstitution, the as-received formulation powder was treated as unknown sample and its ATR spectrum was recorded one month after the construction of the calibration line. The PIP concentration was found to be (88.68 ± 0.39)% *w*/*w* and that of TAZ was equal to (11.32 ± 0.39)% *w*/*w*. The relative error values of the results mentioned above were found to be 0.36% and 2.91%, respectively.

## 3. Discussion

The aim of the present study was to apply the sample preparation method of film formation, on the ATR crystal after solvent evaporation, in order for piperacillin and tazobactam, two different APIs coexisting in a commercial drug mixture, to be identified and quantified. The above- mentioned methodology proved to be a valuable tool that exceeded our expectations. This sample preparation technique ensured a good contact between the sample and the ATR crystal, thus making it possible to enhance the spectral detail. The amount of liquid formulation placed on the crystal had such a value that the film formed thereon was thin enough to permit the acquisition of a complete spectral image of the sample. Given that the penetration depth of the IR beam is restricted to a few microns, it was achieved to obtain whole analyte information. Such a sample preparation method can also overcome the usual problems with solvent IR absorption, which are especially significant for aqueous solutions, irrespective of the concentration of analytes dissolved in it. An additional advantage of the technique used is that by handling a liquid sample, no pressure needs to be employed on the sample surface, thus protecting crystal materials prone to crack. 

Although the formulation had a quite low content of TAZ API and the characteristic peaks of the latter were extensively overlapped by those of PIP, it was effectively identified. The quantification of APIs’ concentrations in solid drug took place after selection of a suitable pair of bands, which could yield reliable results. Variations in signal intensity of some characteristic peaks of each API between replicate samples was not only due to non-uniform distribution of analyte particles on the surface of the instrument’s crystal, but also to different orientation of them, thus affecting their interaction with radiation. The calibration curve obtained presents a linear dynamic range from 5% to 50% *w*/*w* TAZ and a correlation coefficient about 0.999. 

Up to date, piperacillin and tazobactam have been in most cases analyzed through chromatographic techniques, which are quite sensitive and precise but gather some important disadvantages. Apart from being time-consuming, HPLC requires large volume of solvents, thus excluding itself from the category of ‘’green’’ techniques. UHPLC, on the other hand, takes only 5 min to be applied and few µL of sample, but the whole procedure still requires a mobile phase and a more specialized user. Also, the proposed methodology can be applied directly to dry formulation before reconstitution i.e., no sample preparation is involved, while chromatographic techniques can be applied only for liquid samples. That was proved when the performance of the calibration line was tested not only for the liquid reconstituted sample after drying on the ATR crystal but also for the solid formulation.

Considering the above, such an analytical technique combined with the proposed relatively simple sample treatment should be considered as an alternative when developing quality-control procedures for solid or liquid pharmaceuticals. The advantages it gathers make it potentially ideal for in-line control in manufacture in order for simultaneous quantification of multiple products to be performed. Besides, the methodology presented here is easy to be applied, not only for this formulation but for numerous intravenous administered formulations containing various APIs such as Vancomycin, Voriconazole, Propofol, since it requires limited training and no particular analytical knowledge. Thus, it can be incorporated in a hospital workflow. The identification and quantification of the drug formulation in such cases could be performed either after reconstitution of the formulation (using a negligible amount of the liquid drug from its glass bottle by syringe) or before its administration (using the liquid drug from the intravenous infusion bag where it would have been further diluted) or even by directly checking the dry formulation before reconstitution. A nurse or the hospital pharmacist could then place either the dry formulation directly on the ATR crystal and obtain with no time delay the spectrum of formulation (no sample treatment is involved) or use the liquid sample after reconstitution and film formation so as to record the spectrum. Such an approach is quite promising as it could confirm the identity as well as the concentration of a formulation before its administration to a patient, thus eliminating errors that are irreversible and can cost human lives. 

## 4. Materials and Methods

In the present study, pure piperacillin and tazobactam APIs, were purchased in the form of sodium salts, from Glentham Life Sciences (Corsham, UK), which is a UK based supplying company. The generic formulation used was Zobactam^®^ (Vocate Pharmaceuticals S.A., Athens, Greece), a combination of piperacillin and tazobactam in a mass ratio of 8:1, respectively. The formulation was in the form of powder for injectable solution with no excipients and had been supplied by Aenorasis S.A., a Greece based medical equipment company (Athens, Greece). In order to complete reconstitution of 4.5 g of the drug to be achieved, 20 mL of water for injection were used. 

### 4.1. Preparation of Standard Mixtures

Pure APIs were used to prepare 6 dry mixtures. The mass ratio of TAZ:PIP ranged from 50:50 to 5:95. Using an analytical balance (Kern Inc., Grove City, OH, USA, ABJ 220-4NM) and depending on the desired ratio, an appropriate amount of each API was obtained in order for a total mass of 100 mg of the standard mixture to be prepared (e.g., for the dry mixture 25:75, 25 mg TAZ and 75 mg PIP were obtained). Each mixture was placed in a special plastic container (NALGENE^®^, Rochester, NY, USA) having a magnetic rod and was homogenized by placing the latter on a magnetic stirrer (HANNA instruments, HI 190M (Woonsocket, RI, USA)) for 5 min. 

### 4.2. Preparation of Solutions of Standard Mixtures

The total mass of the prepared dry mixtures was transferred to 20 mL glass vials and dissolved by adding 2 mL of water for injection. The solvent was added using a 1 mL automatic pipette (Labnet International Inc., Edison, NJ, USA, Biopette^TM^, Autoclavable Pipettes). In order for complete dissolution to be achieved, Vortex (IKA^®^, Staufen, Germany, MS2, Minishaker) as well as ultrasonic bath (Branson Ultrasonics, Danbury, CT, USA, 2510E-MT) at a frequency of 60 HZ were used for about 30 s and 15 min, respectively. 

### 4.3. Development of Film Forming Methodology

**Figure 6:**
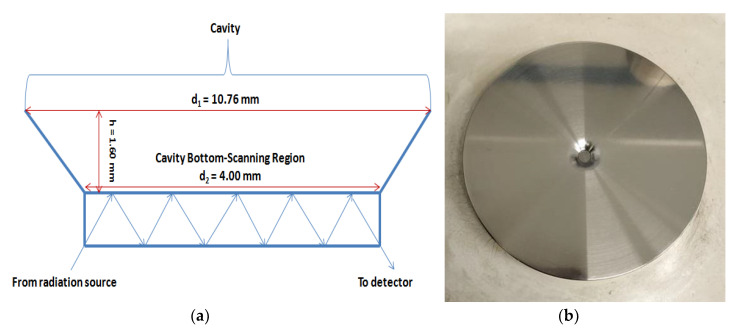
(**a**) Scheme of ATR Instrumentation-Sample Position Structure; (**b**) Photo of ATR Instrumentation-Sample Position Structure.

The instrumentation used was comprised of a cavity, the circular bottom of which had an internal diameter of 4.00 mm, an external diameter of 10.76 mm and a depth of 1.60 mm and was in direct contact with the 9 bounce ATR crystal, as shown in Figure 6. The sample tested was placed in the center of this cavity in order for the radiation to interact with the analyte molecules precipitated thereon after evaporation. Of great significance was the amount of liquid sample placed in the cavity, so as not only intense peaks to be detected and a good signal-to-noise ratio to be achieved, but also no sample particles to be precipitated outside the bottom of it (scanning region). After trials it was concluded that the capacity of the cavity was of about 70 µL and the evaporation time was more than 2 h. An amount of 10 µL of the sample was tested, but it was noticed that not all analyte particles were precipitated within the scanning region and the evaporation time exceeded 30 min. Finally, 5 µL was the sample quantity considered as the most suitable for this methodology. Firstly, due to the fact that it was adequate to cover the whole surface of the scanning region so as more analyte particles to be precipitated thereon and not outside it, thus more intense peaks to be detected. Secondly, the evaporation time, in this case, was equal to 20 min.

### 4.4. FT-IR/ATR Spectra Acquisition

ATR spectra were obtained using a FTIR-ATR spectrometer (PerkinElmer Spectrum 100) with a 9 bounce Diamond/ZnSe crystal (Waltham, MA, USA). The background spectrum within the instrument was recorded prior to the start of each measurement. The powder samples of APIs and the formulation were measured by placing about 30 mg of each on the surface of instrument’s crystal using a spatula. Then, by the aid of the ATR pressure arm, 60% of the total pressure applicability was used so as good contact between analyte molecules and the crystal to be achieved. The pressure applied was constant for all solid samples. As for liquid samples, 5µL of each solution of standard dry mixtures was placed on ATR crystal using a 10 µL automatic pipette (Scilogex, MicroPette Plus Autoclavable pipettor, YM3K012623) and the spectrum of the sample film was acquired 20 min later, as soon as the solvent had been evaporated. This was repeated for 3 different solution portions from each sample. Each measurement was the result of 25 scans in the mid-infrared region (4000–600 cm^−1^) and instrument’s resolution was equal to 4 cm^−1^.

### 4.5. Spectra Processing 

Spectral data were processed using Origin (OriginPro8, OriginLabPro^®^, Northampton, MA, USA) and recorded as absorbance data. OriginPro8 integration method of measuring the absolute height (or intensity) of selected peaks was used in conjunction with different combinations of fingerprint APIs’ peaks so as to assess the potential to quantify the level of APIs present. The calibration graph was prepared not for individual peaks, but for a combination of them. The one and only baseline corresponding to both of the neighboring peaks of interest (873 cm^−1^ for TAZ and 890 cm^−1^ for PIP) ranged from 862 to 905 cm^−1^.

## 5. Conclusions

Identification and quantitative determination of intravenously administered TAZ-PIP formulation, before and after reconstitution, was accomplished successfully by FT-IR/ATR. The initial powder formulation was analyzed by placing the powder directly on the ATR crystal, while the resulted solution, after reconstitution, was analyzed after the evaporation of the water and the subsequent film formation on the ATR crystal, using less than 2 µL. The latter approach, despite being more time-consuming, due to required time for the film formation, has the advantage of being aseptic and practically non-destructive for the sample since the minute amount needed for the measurement can be drawn with a syringe directly from the bottle or from the infusion bag. Usage of FT-IR/ATR, through film formation, is less complicated than most of the other analytical techniques and thus, this methodology can be introduced into the workflow of a hospital for the reliable, easy to apply, quantitative, and qualitative analysis of all intravenously administered formulations. 

## Figures and Tables

**Table 1:** Vibration modes of some prominent peaks of pure active pharmaceutical ingredients (APIs).

API	Wavenumber (cm^−1^)	Vibration Mode
PIP	728, 762, 850, 890	Aromatic CH wagging
	1072, 1094	In-plane movement of aromatic carbons
	1461, 1602	Semi-circle stretching and Quadrum stretching of aromatic ring
	1713, 1764	Carbonyl group (CO) stretching
TAZ	1135, 1191, 1312	In-phase and Out-of-phase stretching of C-SO_2_-C
	1232, 1452	N=N stretching
	1385, 1616	Carbonyl group (CO) stretching

**Table 2:** Spectral differences between PIP and formulation FT-IR/ATR spectra obtained from powder samples of the substances, due to TAZ presence.

Wavenumber (cm^−1^)	Observation
873	Intensity enhancement and change in intensity ratio against the characteristic PIP peak at 890 cm^−1^
945	Intensity enhancement and change in intensity ratio between neighboring peaks at 933 and 949 cm^−1^ (area A)
1023	Intensity enhancement and change in intensity ratio against the neighboring peak at 1007 cm^−1^
1081	Intensity enhancement and change in intensity ratio between neighboring peaks at 1072 and 1094 cm^−1^ (area B)
1135	Detection of TAZ shoulder

**Table 3:** Mean peak height ratio, *I*(873)/*I*(890) and relative standard deviation (RSD) values corresponding to the three replicate film measurements, for each one of the standard mixtures tested.

% Mass Ratio TAZ-PIP	*I*(873)/*I*(890)	RSD%
50:50	2.925	3.14
45:55	2.493	2.19
25:75	1.138	3.48
15:85	0.699	2.22
10:90	0.517	3.89
5:95	0.367	1.90

## Appendix A

Appendix A is a section containing the mathematical proof of how the final equation of the calibration curve was developed. Spectral observations arising from film spectra of both pure active substances and the commercial formulation (Figure 1 and Figure 2) confirmed that peak at 873 cm^−1^ is a result of not only PIP but also TAZ presence. Thus, signal intensity at that position is expected to be given from the Equation:

(A1) $$I\;\left(873\;{\mathrm{cm}}^{-1}\right)=k_{1}\times C_{PIP}+k_{2}\times C_{TAZ},$$

where $k_{1}$ and $k_{2}$ are standards correlating the value $I\;\left(873\;{\mathrm{cm}}^{-1}\right)$ with $C_{PIP}$ and $I\;\left(873\;{\mathrm{cm}}^{-1}\right)$ with $C_{TAZ}$ respectively.

Due to the fact that peak at 890 cm^−1^ is characteristic of piperacillin, its intensity is expected to be given from the equation:

(A2) $$I\;\left(890\;{\mathrm{cm}}^{-1}\right)=k_{3}\times C_{PIP},$$

where $k_{3}$ is a standard correlating the value $I\;\left(890\;{\mathrm{cm}}^{-1}\right)$ with $C_{PIP}$.

If (A1) is divided by (A2), then it turns out that:

(A3) $$\frac{I\;\left(873\;{\mathrm{cm}}^{-1}\right)}{I\;\left(890\;{\mathrm{cm}}^{-1}\right)}\;=\;\frac{k_{1}}{k_{3}}\;+\;\;\frac{k_{2}}{k_{3}}\;\times \;\frac{C_{TAZ}}{C_{PIP}}$$

Given that the formulation consists exclusively of the APIs it is valid that:

(A4) $$C_{TAZ}=100-C_{PIP},$$

Finally, combining (A3) with (A4) a new equation is formed, where:

(A5) $$\frac{I\;\left(873\;{\mathrm{cm}}^{-1}\right)}{I\;\left(890\;{\mathrm{cm}}^{-1}\right)}\;=\;\frac{k_{1}-k_{2}}{k_{3}}\;+\;\;\frac{k_{2}}{k_{3}}\;\times \;\frac{100}{C_{PIP}}$$
